# Ultra-low binder content 3D printed calcium phosphate graphene scaffolds as resorbable, osteoinductive matrices that support bone formation in vivo

**DOI:** 10.1038/s41598-022-10603-3

**Published:** 2022-04-28

**Authors:** Leila Daneshmandi, Brian D. Holt, Anne M. Arnold, Cato T. Laurencin, Stefanie A. Sydlik

**Affiliations:** 1 Connecticut Convergence Institute for Translation in Regenerative Engineering, UConn Health, Farmington, CT 06030 USA; 2 Raymond and Beverly Sackler Center for Biological, Physical and Engineering Sciences, UConn Health, Farmington, CT 06030 USA; 3 Department of Biomedical Engineering, University of Connecticut, Storrs, CT 06269 USA; 4 Department of Orthopaedic Surgery, UConn Health, Farmington, CT 06030 USA; 5 Department of Chemistry, Carnegie Mellon University, 4400 Fifth Avenue, Pittsburgh, PA 15213 USA; 6 Department of Material Science and Engineering, University of Connecticut, Storrs, CT 06269 USA; 7 Department of Chemical and Biomolecular Engineering, University of Connecticut, Storrs, CT 06269 USA; 8 Department of Biomedical Engineering, Carnegie Mellon University, 5000 Forbes Avenue, Pittsburgh, PA 15213 USA; 9 Present Address: National Security Directorate, Pacific Northwest National Laboratory, Richland, WA 99354 USA

**Keywords:** Biomedical engineering, Implants, Bioinspired materials, Biomaterials - cells, Implants, Tissues, Graphene

## Abstract

Bone regenerative engineering could replace autografts; however, no synthetic material fulfills all design criteria. Nanocarbons incorporated into three-dimensional printed (3DP) matrices can improve properties, but incorporation is constrained to low wt%. Further, unmodified nanocarbons have limited osteogenic potential. Functionalization to calcium phosphate graphene (CaPG) imparts osteoinductivity and osteoconductivity, but loading into matrices remained limited. This work presents ultra-high content (90%), 3DP-CaPG matrices. 3DP-CaPG matrices are highly porous (95%), moderately stiff (3 MPa), and mechanically robust. In vitro, they are cytocompatible and induce osteogenic differentiation of human mesenchymal stem cells (hMSCs), indicated by alkaline phosphatase, mineralization, and COL1α1 expression. In vivo, bone regeneration was studied using a transgenic fluorescent-reporter mouse non-union calvarial defect model. 3DP-CaPG stimulates cellular ingrowth, retains donor cells, and induces osteogenic differentiation. Histology shows TRAP staining around struts, suggesting potential osteoclast activity. Apparent resorption of 3DP-CaPG was observed and presented no toxicity. 3DP-CaPG represents an advancement towards a synthetic bone regeneration matrix.

## Introduction

Bone biomaterial matrices aim to support osseous tissue regeneration at the defect site while degrading and being replaced with newly generated bone. Traditional bone biomaterials were developed to replace the lost tissue through simply filling the defect and stabilizing the injury; contemporary materials additionally aim to be biologically active and regenerate functional bone tissue^1^. Despite advances towards this aim, there is still no material to replace autogenous bone and possess all the desirable properties including osteoinductivity, biological safety, a long-shelf life, and reasonable production costs^2^.

A number of materials have been applied to produce a synthetic matrix that meets these design criteria^3–5^. Among these, graphenic materials have received considerable attention due to excellent chemical, mechanical, and biological properties, including tunable functionality, strength, and intrinsic compatibility. These nanostructured carbon materials have a myriad of desirable characteristics that make them promising for use in synthetic matrices. Pristine graphene is comprised of sp^2^ hybridized carbons with delocalized π electrons. This chemically resilient structure is exceptionally strong^6^ and has a large specific surface area^7^ that is useful for physically reinforcing matrices. Strong oxidation reaction conditions can convert some sp^2^ hybridized carbons into sp^3^ hybridization. This oxidized form, known as graphene oxide (GO), possesses oxygen chemical handles for processability and functionalization^8,9^. Further, disruptions to the π–π bonding of pristine graphene dramatically increases the susceptibility of the oxidized backbone to further chemical reactions. Thus, GO is degradable through biologically relevant conditions and follows both an aqueous degradation pathway^10,11^ and an enzymatic and oxidative biodegradation pathway^12–18^, enabling application as a degradable matrix. From a biological standpoint, graphenic substrates can promote the adhesion and growth of cells, with further evidence suggesting their osteogenic potential^19,20^. Because of this, graphenic materials applied as biomaterials have expanded rapidly, especially for bone regenerative engineering^21–23^.

We previously reported the creation of an intrinsically osteoinductive family of functional graphenic materials termed phosphate graphenes (PGs) with potential for bone regeneration^24–26^. PGs are prepared through a synthetic modification of GO in which polyphosphates are covalently installed onto GO and balanced with a variety of counterions (e.g., Ca^2+^, K^+^, Li^+^, Mg^2+^, or Na^+^). Through controlling the identity of the counterions and the agility of the synthetic method, we can readily tune the properties of PGs, including their surface chemistry, degradability, mechanical properties, bioactivity, and osteoinductivity. The bone mineral-mimicking calcium PG (CaPG) elutes Ca^2+^ and PO_4_^3−^ inducerons that serve as signaling molecules to induce osteogenesis^27,28^. We demonstrated that CaPG is inherently osteoinductive, driving the differentiation of stem cells into bone cells, and, when combined with bone marrow stromal cells (BMSCs), it induces de novo osteogenesis ectopically in the subcutaneous space of mice^24^. Importantly, CaPG is degradable into cytocompatible products through both of the major in vivo degradation pathways: hydrolytic and enzymatic^25^. While the powder slurry application of CaPG in our previous study may be suitable for non-load bearing sites, a mechanically robust CaPG matrix that provides the mechanical and biochemical stimuli in a spatiotemporal manner is necessary to make this technology clinically viable.

Additive manufacturing enables the generation of customizable, complex three-dimensional (3D) structures. Recently, graphenic materials have been incorporated in additive manufacturing processes to enhance the osteogenic properties of the resulting matrices for bone regeneration^29,30^. However, unfunctionalized graphenic materials lack osteoinductivity, and inclusion of graphenic materials into 3D printed matrices is typically limited to a few wt%^31^ or non-covalent coating of the surface after fabrication. In the literature, most matrices are not investigated in vivo, although some papers report encouraging preclinical animal results^32–36^. Overviews of this emerging area are provided in two contemporary reviews^29,30^.

To achieve an intrinsically osteoinductive, customizable matrix primarily composed of functional graphenic materials, we developed and studied the osteogenic potential of 3D printed CaPG (3DP-CaPG) matrices in vitro and in vivo. We employed the services of a commercially available 3D printing method to print porous constructs of our CaPG with high weight fraction (90% w/w). This method enables a remarkably high graphenic content within the ink, readily enabling cellular access to the osteoconductive backbone and controlled release of calcium and phosphate ions from the osteoinductive functionalization. Thus, host response to the matrix is dominated by the functional graphenic content and not the bioinert binder. Our matrices show osteogenic efficacy both in vitro with human mesenchymal stem cells (hMSCs) and in vivo in an orthotopic bone defect model. Osteomimetic CaPG matrices are mechanically resilient, biocompatible, biodegradable, osteoconductive, and osteoinductive, potentially disrupting the paradigm in bone regeneration.

## Results and discussion

### Material design

Bone has the innate capacity to regenerate small fractures and remodel tissue throughout the lifetime of an organism. Natural bone undergoes a constant remodeling process in which osteoclasts resorb tissue, following which osteoblasts rebuild bone. In cases of severe injury, this natural process is disrupted due to a loss of scaffolding to guide regenerating tissue. Thus, it is essential that a matrix is introduced at the site of a critical injury to restore physiological function. Matrix morphology, composition, and biodegration rate are important design criteria for osteogenic regenerative medicine^37–39^. Traditional synthetic bone matrices, however, are biologically inert and non-resorbable, remaining with patients throughout their lifetimes. Hence, there is a need to create superior, synthetic matrices to treat severe bone injuries that both support the healing tissue and facilitate the innate resorption and reconstruction process.

To fulfill this need, we designed a superior synthetic bone matrix capable of tissue regeneration. Our design utilized CaPG, a functional graphenic material functionalized from GO. GO already meets several criteria for an ideal synthetic bone matrix: resorbability, biocompatibility, and osteoconductivity. However, GO lacks the chemical cues necessary to initiate the regeneration of native bone at the site of injury. Further, while GO flakes have excellent mechanical properties, bulk constructs of GO lack the water stability necessary to provide mechanical support for regenerating bone.

**Figure 1:**
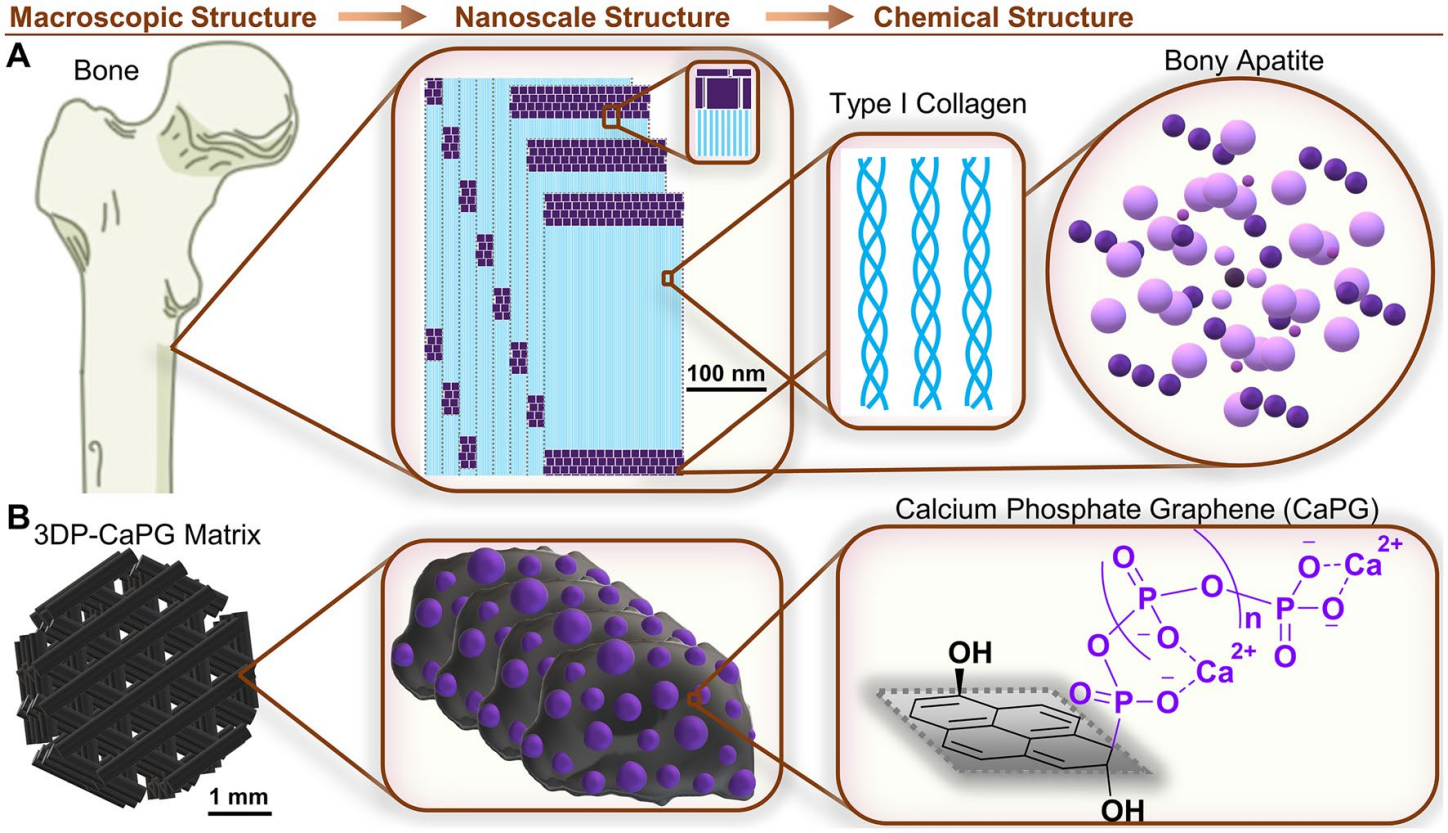
Biomimetic 3D printed CaPG matrix design. (**A**) Illustrative example of the structure of bone at the indicated length scales. (**B**) Illustration of the corresponding 3D printed CaPG matrix structure. Note that the graphenic sheet of CaPG is shown as a pyrene structure for simplicity.

The capacity for bone to regenerate is a consequence of the mechanical and biochemical cues that are orchestrated in an accurate spatiotemporal manner. Specifically, the base components of bone tissue are type I collagen and bone mineral primarily consisting of carbonated hydroxyapatite^40–48^ (Fig. 1A). The bulk structure of bone tissue serves as a matrix to guide regenerating tissue, which is known as osteoconduction. Further, the phosphate and calcium of the bone mineral play an integral role in the initiation of bone regeneration, known as osteoinduction, in natural healing processes^28,49–62^.

Here, we used a biomimetic approach to chemically modify GO into a synthetic material that promotes bone regeneration: CaPG^24,25^. In CaPG, polyphosphates are covalently installed on the surface of GO in a “grafting from” approach, while calcium ions are incorporated through electrostatic interactions with negatively charged phosphate groups (Fig. 1B, Supplementary Figs. 1, 2).

We previously demonstrated that CaPG retains the desirable properties of GO—including hydrolytic and enzymatic degradability^11,25^, biocompatibility^24,63^, and osteoconductivity^11,24^—while the inclusion of phosphate and calcium imparts osteoinductive properties^24^. CaPG possesses innate osteoinductivity; however, for CaPG to become clinically viable, it needs to be processed into customizable, mechanically robust matrices that recapitulate the spatiotemporal regenerative qualities of bone.

3D printing enables on-demand generation of materials with complex structures. Although most 3D printing technologies are incompatible with high graphenic content^64,65^, a commercially available direct ink writing option is available through Dimension Inx that can 3D print matrices with up to 90% graphenic content and the rest biocompatible, biodegradable, elastic poly(lactic-*co*-glycolic acid) (PLGA) binder^64–66^. The graphenic ink (90 wt% graphenic content and 10 wt% PLGA) is room temperature extruded to produce self-supporting structures without additional crosslinking or sintering^64^. The PLGA binder is FDA-approved for biomedical applications, and Dimension Inx’s 3D printing technology has been shown to be biocompatible for osteogenic regenerative medicine applications^65^.

**Figure 2:**
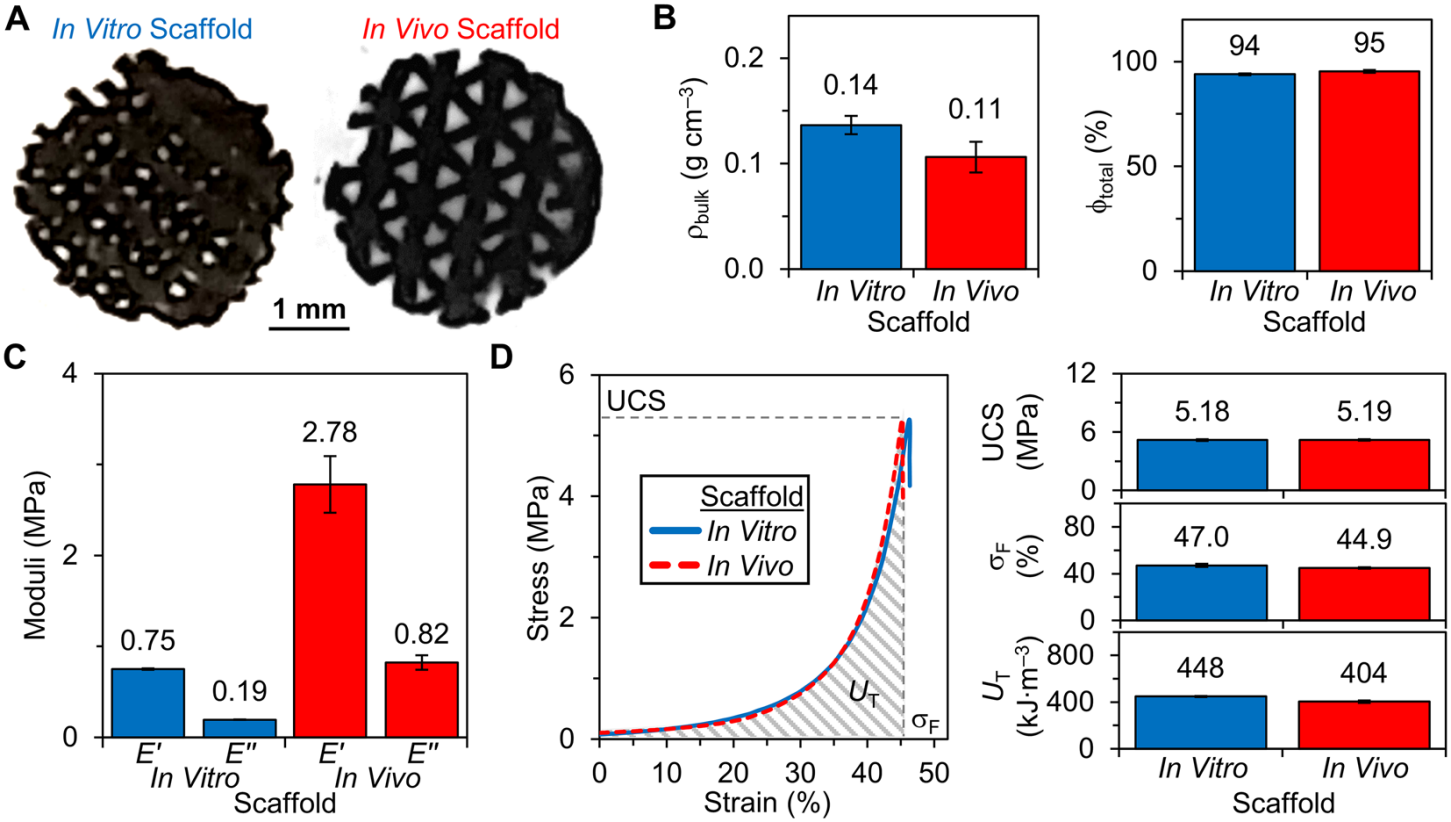
Bulk and mechanical properties of 3D printed CaPG matrices for in vitro and in vivo use. **(A)** Images of the in vitro and in vivo matrices. **(B)** The bulk density (ρ_bulk_) and total porosity (ϕ_total_) of matrices. **(C)** The compressive storage (*E'*) and loss (*E''*) moduli of matrices. **(D)** The stress–strain curves and their quantified parameters: ultimate compressive strength (UCS), strain at failure (σ_F_), and toughness (*U*_T_) of matrices. Note that bars in (**B–D**) are the average of *n* = 3 measurements, and error bars are standard deviation.

Thus, we sent our CaPG powder to Dimension Inx and paid it to 3D print large sheets of porous CaPG matrices. We purchased sheets with identical print parameters other than layer offset (90° and 120°), as Dimension Inx—through its own propriety research and development—has identified 90° and 120° offsets to work well for these types of matrices for in vitro and in vivo studies, respectively (Supplementary Fig. 3). The in vitro offset enables more uniform cell seeding while the in vivo offset offers better host integration. Dimension Inx has shown that for high content graphenic inks that this set of print parameters generates matrices with desirable mechanical properties, electrical conductivity, cytocompatibility, bioactivity, biocompatibility, and surgical handling^64,65^. From these large sheets, we could readily cut out matrices with specific geometries for cellular and animal studies (Fig. 2A). Alternatively, matrices may also be able to be directly printed to match a defect. Combining 3D printing with intrinsically osteoinductive CaPG creates a customizable matrix system for bone regenerative engineering.

### Matrix bulk and mechanical properties

A synthetic bone matrix that promotes bone regeneration requires porosity to encourage cell infiltration, while maintaining robust mechanical properties to support the surrounding tissue. Here, the 3D printed CaPG (3DP-CaPG) matrices have a high total porosity (ϕ_total_), up to 95%, and low bulk density (ρ_bulk_), down to 0.11 g cm^–3^ (Fig. 2B). These values are similar to those of trabecular bone, which has a porosity of 50–90% and a density of ~ 0.2 g cm^–3^^67^. The combination of a high ϕ_total_ with a low ρ_bulk_ creates a matrix with a large specific surface area for donor cell attachment and host cell infiltration.

Macroscopic optical images show highly absorbing matrices resulting from 90% composition of CaPG (Fig. 2A). As recommended by Dimension Inx based on its expertise with graphenic matrices, the layer offset was 90° for in vitro and 120° for in vivo testing, with all other parameters the same. During Dimension Inx’s development of this 3D printing technology, it performed scanning electron microscopy that revealed that shear forces during the print processes result in graphene flake alignment along the exterior of the fibers and flake stacking within the fibers^64^. These images were unable to show polymer/graphene interactions, and electron microscopy of polymer/nanocarbon assemblies is challenging^68–70^. Functionalization of the graphenic backbone with polyphosphates likely enables chain entanglements between CaPG flakes that increase the mechanical properties of the matrices. Entanglement may be enhanced by tuning the synthesis of CaPG to increase polyphosphate degree of polymerization and seeding density^25^. Dimension Inx’s patented^66^, 3D printing process includes a high vapor pressure solvent that rapidly evaporates to create self-supporting fibers and low vapor pressure solvents that impart enough liquidity to enable seamless merging of layers^64^.

The compressive mechanical properties of 3DP-CaPG matrices include storage moduli on the order of 1 MPa and strengths of ~ 5.2 MPa (Fig. 2C,D). Slight differences in mechanical properties of matrices for in vitro and in vivo applications are likely due to differences in matrix heights and print offsets. These mechanical properties are impressive for such highly porous matrices and are relatively similar to the Young’s modulus (50–500 MPa) and strength (2–12 MPa) of cancellous bone^67^. However, they are substantially less than the Young’s modulus (7000–30,000 MPa) and strength (100–230 MPa) of cortical bone^67^. Revising the print design could be used to improve mechanical properties to more closely match those of cortical bone. For example, we expect that a matrix with lower porosity would be stronger. Likewise, cortical bone is much denser, with a porosity of 3–12% and a density of 1.80 g cm^–3^^67^.

### In vitro osteogenic differentiation

An intrinsically osteoinductive regenerative bone graft substitute should promote hMSC attachment, viability, and osteogenic differentiation, while maintaining its mechanical properties. To begin to study the biological response, the in vitro compatibility and osteogenic differentiation of hMSCs on 3DP-CaPG matrices were investigated.

**Figure 3:**
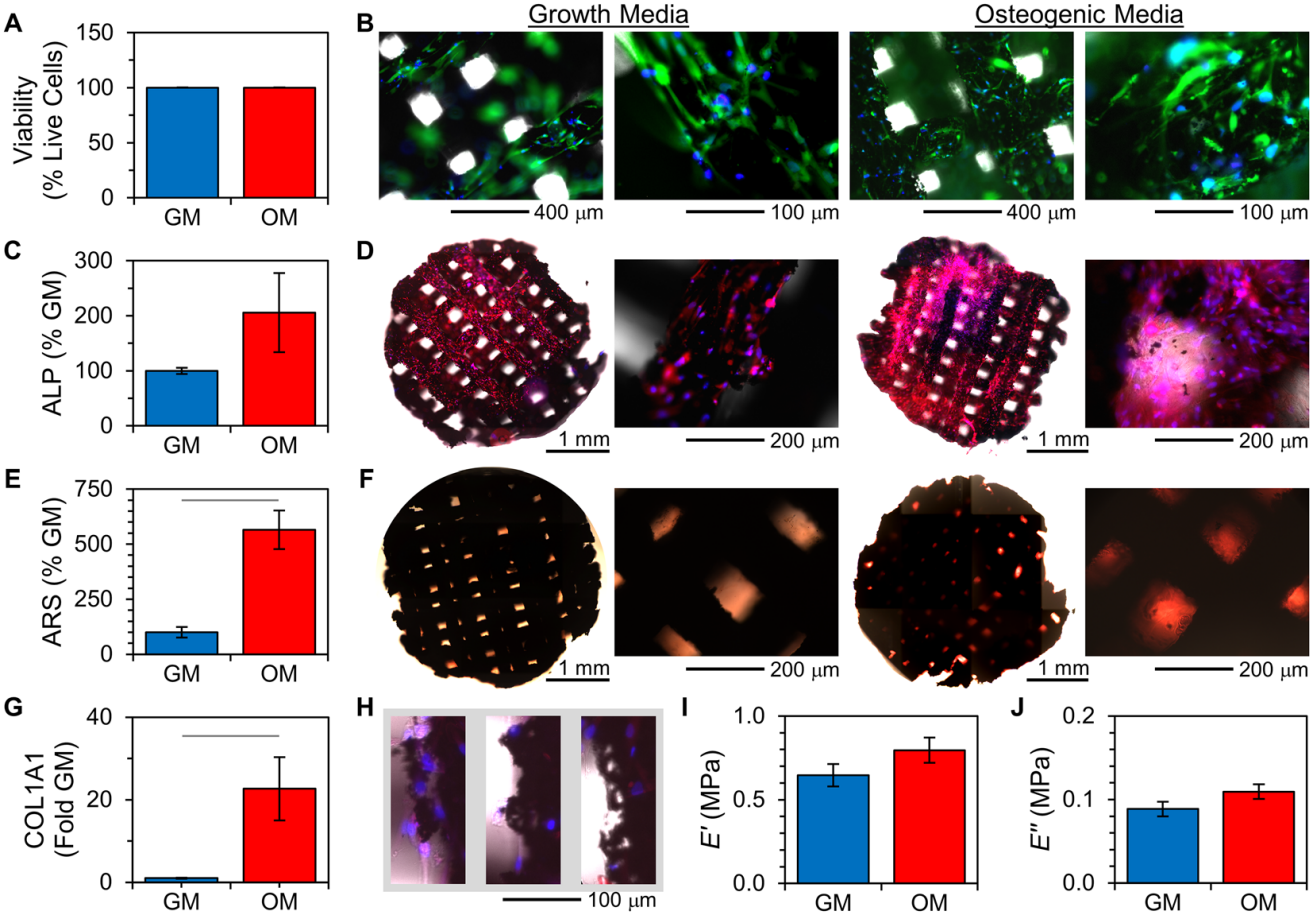
Compatibility and osteogenic differentiation of hMSCs on 3DP-CaPG matrices. **(A)** Percent cellular viability. *n* > 740 cells per condition; *p*-value = 1; error bars are standard deviation of sample proportion and are small (~ 0.1%). **(B)** Cytocompatibility images at different magnifications. Blue is all nuclei (Hoechst 33342); green is metabolically active cells (Calcein AM); and red is nuclei of dying cells (propidium iodide). **(C)** Alkaline phosphatase (ALP) expression, relative to that of hMSCs on matrices cultured in growth media (GM). n = 3 matrices; p-value = 0.22. **(D)** Whole-matrix and higher magnification images of fluorescently labeled ALP (red) and nuclei (blue). **(E)** Calcium deposit quantification using alizarin red S (ARS) labeling. n = 3 matrices; p-value = 0.007. **(F)** Color images of matrices labeled with ARS. **(G)** Relative gene expression of COL1A1 quantified from RT-qPCR. *n* = 3 samples from RNA pooled from cells cultured on 3 separate matrices per condition; *p*-value 0.03. **(H)** Images of struts of 3DP-CaPG matrices. Blue is nuclei; red is ALP; gray is brightfield. **(I–J)** Compressive dynamic mechanical analysis determination of the **(I)** storage (*E'*) and **(J)** loss (*E''*) moduli of the matrices after 10 days of hMSC growth. *n* = 4 matrices; *p*-values = 0.21 and 0.17, respectively. Statistically significant differences are indicated by a line between data bars; unless otherwise indicated, error bars are standard error of the mean.

Matrices were directly seeded with cells, and hMSCs rapidly adhered to and spread over the matrices. After 10 days in culture, hMSCs covered the entirety of the struts throughout the 3D structure and were highly viable (Fig. 3A,B). No differences in cellular vitality were observed in growth media or osteogenic media, and no dying cells were observed.

The hMSCs that were cultured on the 3DP-CaPG matrices underwent osteogenic differentiation, independent of any cues beyond the matrix itself. Cells maintained in both growth and osteogenic media showed a pronounced, intense presence of alkaline phosphatase (ALP) (Fig. 3C,D). ALP is highly expressed in osteoblasts^71^, and its level of staining reflects the degree of osteoblastic expression. Also, there was notable Alizarin Red S (ARS) staining (Fig. 3E,F), which labels calcium deposits that are indicative of mineralization from cells displaying an osteogenic phenotype^72^. The expression of COL1A1, which encodes the major component of type I collagen, was detected for hMSCs cultured in both types of media but was significantly higher (23-fold) for cells cultured in osteogenic media (Fig. 3G).

The high viability of hMSCs grown on the 3DP-CaPG matrices further confirm the compatibility of CaPG. Optical micrographs demonstrate that the matrix struts appeared to be undergoing degradation concomitant with the presence of cells (Fig. 3H). Not only is this finding consistent with the design criteria of a matrix for regenerative engineering, but also the presence of exfoliated CaPG material likely provides additional biomechanical osteoconductive cues as well as enhanced release of Ca^2+^ and PO_4_^3−^ osteoinductive inducerons. In agreement with our previous findings^24^, cellular exposure to CaPG was sufficient to induce osteogenic differentiation. In addition to induceron release, the local stiffness of the micron-sized flakes^73^ may enhance osteogenic differentiation^74^. ALP expression was similar between cells maintained in growth media designed to preserve the potency of hMSCs and cells maintained in osteogenic media formulated to achieve osteogenic differentiation. After 10 days of culture on the matrices, hMSCs cultured in osteogenic media showed 565% stronger ARS labeling than hMSCs cultured in growth media. This significant increase is likely due to osteogenic media driving hMSCs towards an osteoblast phenotype faster than those cells without the additional chemical cues. Additionally, the images of the ARS staining suggest that ARS is labeling calcium deposits produced by the cells and not the matrices. This conclusion is supported by the differences in intensity between the two media types and the fact that the most observable labeling is from spaces proximal to, but not coincident with, the matrix struts.

The mechanical properties of the 3DP-CaPG matrices after 10 days of hMSC culture were similar between types of media (Fig. 3I,J). The storage modulus (*E'*) of the matrices with hMSCs was similar (96 ± 7%) to that of the pristine matrix; however, the loss modulus (*E''*) was lower (51 ± 7%) than that of the pristine matrix. The loss modulus may be decreased due to loss of flexibility as calcium and phosphate ions are released from the polymeric linkers. The cellular biomechanics of adhered cells could also contribute to this change. Overall, the mechanical properties of the matrices remained robust throughout cell culture, even with the observed degradation.

### In vivo osteogenic differentiation

While in vitro data is necessary, it is not always predictive of the in vivo biological response. Thus, we selected a mouse critical-sized calvarial defect model to evaluate the osteogenic efficacy of the 3DP-CaPG matrices in an orthotopic bone site. We designed the experiment to include three groups: 3D printed graphene (3DP-G), 3DP-CaPG, and demineralized bone matrix (DBM), each tested with and without exogenous bone marrow stromal cells (BMSCs). We selected 3DP-G and DBM as control materials. In 3DP-G, the graphenic component is unfunctionalized graphene, allowing us to elucidate the role of graphene independently from inducerons. DBM is the retained organic matrix of allograft bone after the inorganic component is removed and is osteoconductive and has osteoinductive potential^75^. DBM source, processing steps, and carrier vary vastly affecting osteoinductive properties, and for spinal fusion surgery alone there are over 50 commercially available DBM-based products^75^. We chose OsteoWrap demineralized cortical plate membrane as a control because it matched the defect site tissue type and is widely studied in the repair of critical-sized calvarial defects, especially when combined with stem cells^76,77^. Thus, it served as a compatibility control and baseline against which 3DP-CaPG osteoinductive performance could be measured.

The materials were tested in a transgenic fluorescent reporter mouse model as previously reported^24,78^. The Col3.6 fluorescent protein reporter mice contain a 3.6-kilobase DNA fragment derived from the rat type I collagen (Col1α1) promoter that drives strong expression of fluorescent proteins in pre-osteoblasts and osteoblasts. The intensity of the fluorescent protein reflects the level of osteogenic differentiation, with osteoprogenitors expressing low levels and matured osteoblasts expressing higher levels^79^. In this work, we used donor BMSCs from Col3.6Cyan mice and Col3.6Topaz mice as hosts to delineate the contributions of host and donor cells in the regenerative process.

The critical-sized calvarial defect model is a standardized non-union to test bone replacement materials, which is incapable of healing on its own^78,80^. We have previously demonstrated the critical size of this model and its inability to bridge the defect gap in a similar transgenic mouse model in which an unaided and empty defect simply formed a thin layer of fibrous connective tissue and no new bone^78^. The unresponsive environment of the calvarial defect model secondary to its limited endogenous regenerative potential and self-repair capabilities impose a regenerative response that is mostly driven by the contributions of donor cells^81–83^ in synergy with the implant material.

**Figure 4:**
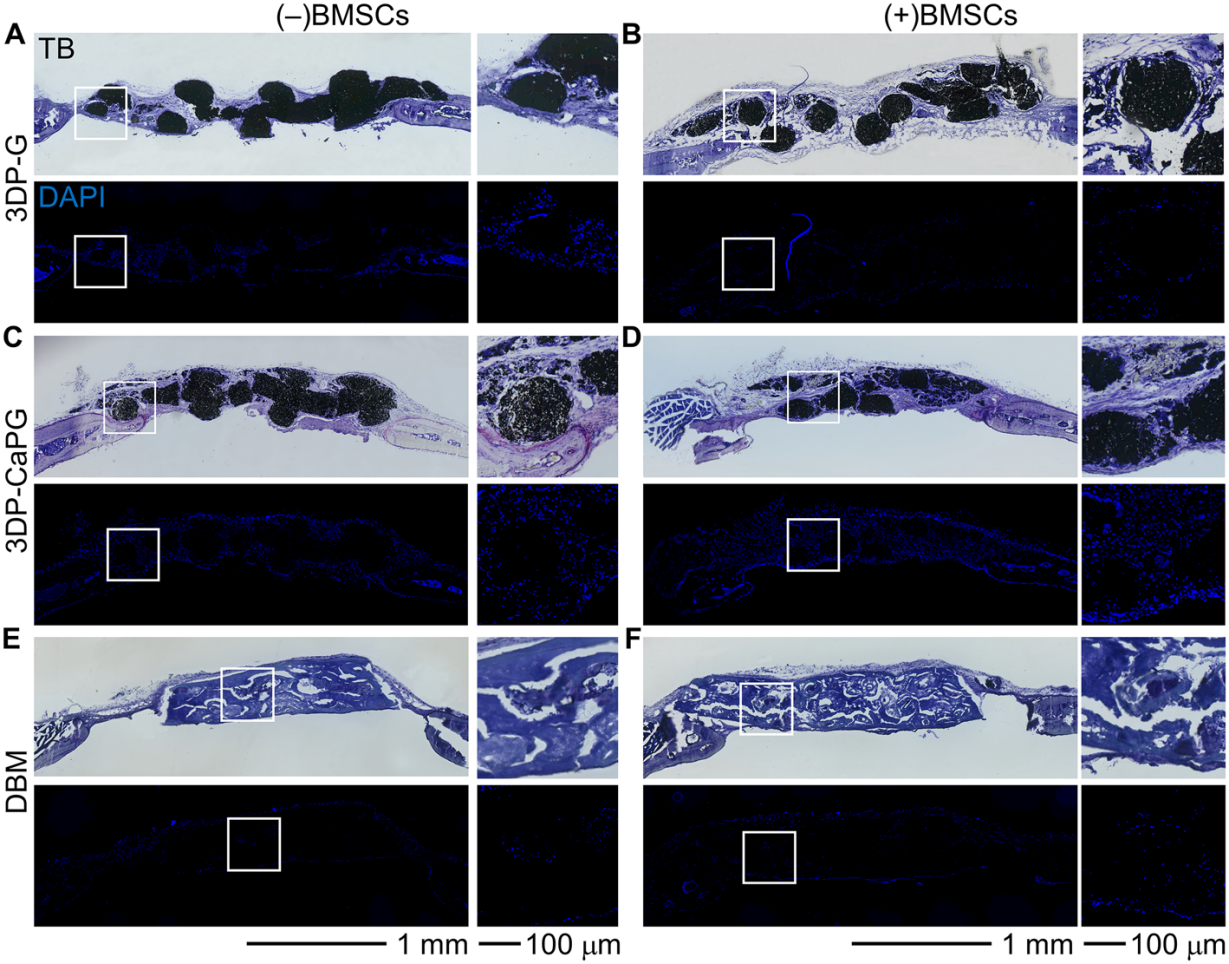
Biocompatibility of the matrices in a mouse calvarial defect model. Histology images of calvarial defects implanted with 3DP-G, 3DP-CaPG, and DBM that are stained with toluidine blue (TB) and DAPI (cell nuclei, blue).

After 8 weeks, the ability of the matrices to regenerate cranial tissue within this critical-sized calvarial defect was analyzed. Toluidine blue (TB) staining of the 3DP-G and 3DP-CaPG defects showed no obvious tissue necrosis or toxicity (Fig. 4A–D). Cellular infiltration and fibrous tissue ingrowth within the 3DP matrices with fibroblastic cells having a linear morphology resembling the morphology found in non-inflammatory environments suggested lack of an inflammatory response. Both 3DP-G and 3DP-CaPG matrices supported host cell migration from the edges of the defect tissue toward the matrices and inward, indicative of their biocompatibility in the host environment. The porosity imparted by the 3D print design and the favorable graphenic material for cellular attachment facilitated cellular infiltration. In the DBM group, cellular migration was mainly confined to implant periphery and its exterior. Lack of infiltration was expected, as this DBM matrix is a cortical plate and primarily serves as a biocompatibility control. For 3DP-G and especially 3DP-CaPG, the presence and distribution of cells was higher than that of DBM, concomitant with the greater host-derived fibrous tissue ingrowth. The greater cellularity in 3DP-CaPG was further corroborated with DAPI-stained cell nuclei of the sections.

**Figure 5:**
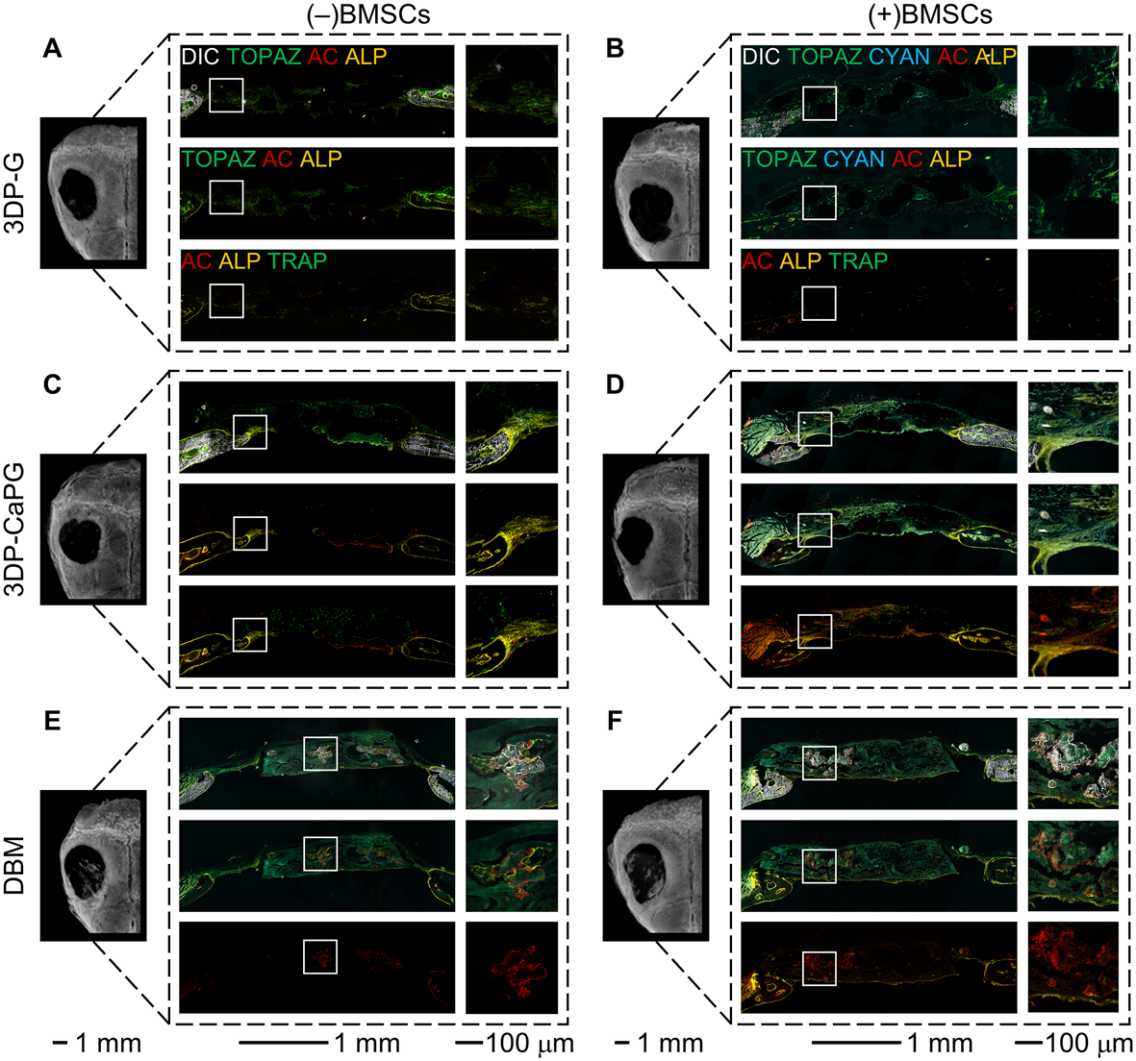
Osteogenic capabilities of 3DP CaPG matrices in a mouse critical size calvarial defect model. Histology overlay images of differential interference contrast (DIC) (grayscale, white mineralized tissue), donor BMSCs (cyan), host cells (topaz), AC (red, active mineralization within the past 24 h prior to sacrifice), ALP (yellow, osteoblast activity), and TRAP (green, resorptive activity).

3DP-CaPG showed ALP activity that was co-localized with the ingrowth of host tissue from the defect edges into the implant (Fig. 5C,D). The inward trajectory of host cell migration and the ingrowth of fibrous tissue are necessary to grow new bone. This was accomplished through the ability of 3DP-CaPG to stimulate the migratory potential of host cells. The growth of fibrous tissue around the CaPG matrix (Fig. 4C,D) and ALP staining, which indicates osteoblastic activity (Fig. 5C,D-top panel), imply that early stage bone formation is taking place. Osteogenic differentiation of host and donor BMSCs into an osteoblast phenotype is driven by the calcium phosphate functionalization of the graphenic backbone. At this time point, the bone formation process was still premature and in its early stages, as there was an absence of bright topaz and donor fluorescent signals for host- and donor-derived osteoblasts, and there was no sharp AC label to indicate active mineralization (Fig. 5C,D-middle panel). The presence of tartrate-resistant acid phosphatase (TRAP)-positive cells, in addition to the alizarin complexone (AC) and ALP labels in 3DP-CaPG, denoted the remodeling of the implant. This suggests the possibility of contributions from an active regenerative process that creates and maintains new bone (Fig. 5C,D-bottom panel).

3DP-G shows fibroblastic cells that express faint topaz fluorescence and are negative for ALP, AC, and TRAP (Fig. 5A,B). Despite being seeded in the same concentration and manner onto matrices of similar geometrical features, cyan donor cells were not detected at the implant site. The inability of the unfunctionalized graphene matrix to retain donor cells upon in vivo implantation was also observed in a subcutaneous ectopic mouse model^24^. Since the regenerative response in the calvarial defect model is greatly dependent on the contributions of donor cells, their loss from the 3DP-G matrices significantly contributed to the poor regenerative response. The inability to retain donor cells and the lack of osteogenic cues for the graphenic material control, 3DP-G, highlight the necessity of CaPG functionalization to produce an optimal osteogenic response.

The OsteoWrap demineralized cortical plate membrane control showed minimal regeneration. One of the major drawbacks in using DBM-based material for orthopedic applications is the inconsistent biological activity and osteoinductive efficacy. These inconsistencies are chiefly because of donor profile, processing methods, and choice of carrier^4,75,84^. In our studies, the DBM plates displayed faint topaz and cyan signals that were most likely background autofluorescence from the material (Fig. 5E,F). Clusters of white mineralized tissue were observed within the implants (Fig. 5E,F-high magnification images) that co-localized with strong AC labeling but were ALP-negative with little to no cellularity (Fig. 4E,F). The absence of cellularity and osteoblastic ALP activity suggest that these clusters were in fact residual bone tissue and mineral retained in the DBM matrix. All DBM used in our study was obtained from the same source and sheet of demineralized cortical plate membrane. The observed regions of white mineral accumulation and AC labeling of calcific depositions suggest that during the processing, old bone could have remained within the matrix. These regions were negative for DAPI, ALP, topaz, cyan and TRAP signals, which indicated neither cellularity, nor new bone-forming activity, nor resorbing activity (Fig. 5F). The minor presence of cells in the DBM-cell clusters (Fig. 5E) can be attributed to residual bone cells or bone morphogenetic proteins and matrix that managed to recruit new cells to form nominal bone.

Since the data demonstrated that bone regeneration was in its early stages for the 3DP-CaPG samples and virtually nonexistent for the 3DP-G and DBM samples, there was minimal mineral to generate signal in radiographs. Further, the residual mineral in the DBM samples would inaccurately contribute to the observed radiopacity of the radiographs. Thus, a radiological comparison of bone regeneration at this 8-week time point was not feasible.

Only 3DP-CaPG effectively retained donor cells within the matrices. Donor cell retention is crucial to bone formation since the regenerative response of the calvarial defect model is primarily driven by donor cells^81–83^, guided by the matrix. This enhanced bone formation for the 3DP-CaPG matrices was manifested through increased ALP activity compared to the 3DP-G and DBM samples.

**Figure 6:**
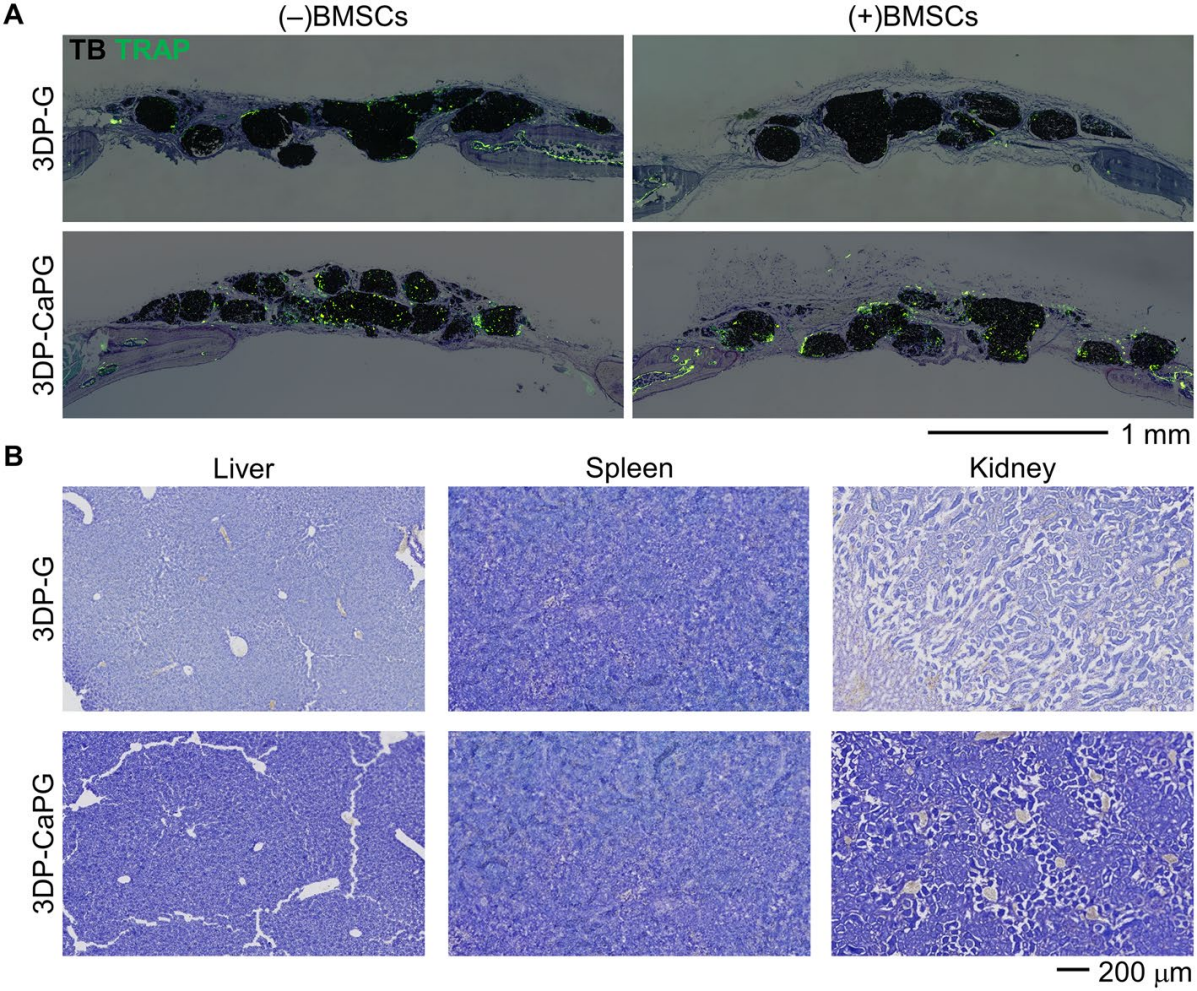
Biodegradation and biodistribution of 3DP-CaPG matrices. **(A)** Histology overlay images of toluidine blue (TB) and TRAP (green, resorptive activity). **(B)** Histology images of the major clearance organs.

Previously, we demonstrated biocompatibility, biodegradation, and intrinsic osteoinductivity of CaPG, but we did not study how these processes synergistically interacted with natural bone remodeling, mediated by osteoclasts^24^. Interestingly, 8 weeks after implantation, we clearly observed less remnant mass of 3DP-CaPG compared to 3DP-G matrices (Supplementary Fig. 4), indicating more rapid biodegradation of the 3DP-CaPG matrices. Substantially greater TRAP-positive staining was observed co-localized with the struts of graphenic material in the 3DP-CaPG matrices compared to the 3DP-G matrices (Fig. 6A). Our previous studies revealed a burst phosphate release due to basal plane scission (~ 84% in 1 day) and a slow, steady release of Ca^2+^ (~ 70% over 28 days) over time in water^25^, inherently aiding resorption of inducerons. The activity of the TRAP-positive cells creates a susceptible environment for CaPG resorption through increasing the acidity, peroxidase enzymatic activity, and general catabolic response. Further, the observation of TRAP-positive cells suggests the possibility for osteoclast-mediated resorption.

The apparent resorption of CaPG prompted us to assess the biodistribution and persistence of the graphenic materials systemically in the mice. We assessed histological sections of the liver, spleen, and kidney, as these are major clearance organs (Fig. 6B). There was no presence or accumulation of any graphenic material in the organs, and there was no obvious tissue damage, structural changes, or pathological damage suggesting the absence of toxicological effects or inflammation imparted by either graphenic material.

## Conclusions

Overall, no commercially available, synthetic bone matrix can completely replace autografts. Using biomimetic, innately osteoinductive CaPG, 3DP-CaPG matrices were fabricated for bone regenerative engineering. These highly porous, 90 wt% CaPG matrices are biocompatible and capable of innately inducing bone regeneration with and without exogenous BMSCs. Notably, CaPG is capable of resorption and biodegradation in vivo, as indicated by increased (+)TRAP cellular activity and observable decreased matrix mass over time. Natural bioresorption is rare for a synthetic material and suggests promise as a resorbable osteoinductive matrix. Further, no detrimental effects or accumulation were observed in vital organs. With additional developmental studies to improve mechanical properties and further confirm long-term safety in vivo, these 3DP-CaPG matrices have potential for bone regeneration applications.

## Methods

### Synthesis of graphene oxide (GO)

GO was synthesized using a method we have previously reported^24,25^. Specifically, GO was synthesized using a modified Hummers’ method^85^. Here, 10 g of graphite flakes (graphite flake, natural, − 325 mesh, 99.8% metal basis; Alfa Aesar, Ward Hill, MA, USA) were dispersed in 250 mL of concentrated sulfuric acid (Fisher Scientific, Pittsburgh, PA, USA) in a 2 L Erlenmeyer flask. The graphite dispersion was placed over ice and cooled. Then, 20 g of KMnO_4_ (Sigma-Aldrich, St. Louis, MO, USA) was slowly added in small aliquots over 20–30 min with stirring while maintaining the ice bath. The ice bath was removed, and the mixture was allowed to warm to room temperature and stirred for 2 h, followed by gentle heating to 35 °C and an additional 2 h of stirring. The heat was then removed, and the reaction was quenched by quickly adding 1400 mL of deionized (DI) water followed by the slow addition of 20 mL of 30% hydrogen peroxide (Fisher Scientific). The remainder of the DI water (450 mL) was slowly added at first until bubbling ceased and then added in one aliquot. The mixture was left to stir at room temperature overnight.

GO was purified via vacuum filtration through a Buchner funnel with large cellulose filter paper. Following filtration, the filtrate was discarded, and the GO was carefully removed from the Buchner funnel without scraping the cellulose filter paper. GO was directly added into 3500 molecular weight cutoff dialysis tubing (SnakeSkin dialysis tubing, Thermo Scientific, Waltham, MA, USA). Then, GO was dialyzed against DI water for 3–5 days until the water was clear. On the first day, the DI water was changed twice and then changed once each day thereafter. The GO was transferred from the dialysis tubing into 50 mL conical centrifuge tubes, with no more than half the tube filled, and frozen to − 80 °C and lyophilized for 3–5 days until dry.

### Synthesis of calcium phosphate graphene (CaPG)

CaPG was synthesized from GO using a modified Arbuzov method that we have reported previously^24,25^. Here, 500 mg of GO, 500 mg of magnesium bromide diethyl etherate (Alfa Aesar, Haverhill, MA, USA), and 500 mL of triethyl phosphite (Sigma Aldrich, St. Louis, MO, USA) were added in sequential order to a 1 L, flame dried round bottom flask under nitrogen. The mixture was bath sonicated (240 W, 42 kHz, ultrasonic cleaner, Kendal) for 1 h to ensure homogeneous dispersion of reagents followed by the addition of 2.5 g of anhydrous calcium bromide (Alfa Aesar, Haverhill, MA, USA). The mixture was then sonicated for an additional 30 min. After sonication, the reaction was heated to reflux (156 °C) under stirring and nitrogen. After a 72 h reflux, the heat was removed, and the reaction was cooled to room temperature and filtered via vacuum filtration through a Buchner funnel containing large cellulose filter paper. The filtrate was discarded and the CaPG filter puck was carefully removed from the funnel without scraping the filter paper and placed into 50 mL conical centrifuge tubes.

CaPG was purified by adding ~ 45 mL of fresh solvent to the centrifuge tube containing the material followed by vortexing and then centrifugation at 3600×*g* for 5 min. Centrifugation pelleted the CaPG to the bottom of the tube, and the supernatant was discarded. The CaPG pellet was re-dispersed in fresh solvent, vortexed, and centrifuged again for the next wash step. Several wash steps were repeated to purify CaPG that included 2 acetone, 1 ethanol, 1 DI water, and 2 acetone washes. The resulting CaPG pellet was then dried under vacuum for 24–48 h until dry.

### Fourier-transform infrared (FTIR) spectroscopy

FTIR spectra were collected on a PerkinElmer Frontier FT-IR Spectrometer with an attenuated total reflectance (ATR) attachment, where the ATR attachment contained a germanium crystal. Raw spectra were recorded in percent transmittance from 4000 to 700 cm^–1^ with a 4 cm^–1^ resolution. ATR and baseline corrections of the raw spectra were performed in Spectrum software (Spectrum version 10, PerkinElmer, https://www.perkinelmer.com/product/software-kit-spectrum-10-lx108873). The data was smoothed with a boxcar of 10 and then offset for clarity.

### Thermogravimetric analysis (TGA)

TGA was conducted on a PerkinElmer TGA 4000 using PerkinElmer ceramic TGA pans. The pans were cleaned, and flame dried prior to all TGA measurements. Further, TGA was performed from 50 to 800 °C with a heating rate of 10 °C/min under a nitrogen atmosphere with a 20 mL min^–1^ flow rate.

### X-ray photoelectron spectroscopy (XPS)

All XPS analysis was conducted on a Thermo Fisher ESCALAB 250 Xi instrument using an Al K-Alpha source gun and a flood gun in charge compensation standard mode. Spectra were acquired using the standard lens mode (angle and field of view of 32,000 steps), Constant Analyzer Energy (CAE) scan mode, and a 200 μm analysis spot size. The graphenic samples were prepared by adhering the powders on double-sided copper tape. Care was taken to ensure that there was no loose powder and that the substrate was completely covered. Then, the double-sided copper tape containing the samples was mounted onto a sample boat for analysis.

Survey spectra were collected for graphenic materials using 5 cumulative scans per spectrum. Further, the survey spectra were acquired over a binding energy range of 1350 to − 10 eV, using a pass energy of 150 eV, an energy step size of 1.0 eV, and a dwell time of 10 ms. The elemental composition of each graphenic material was quantified from the survey spectrum by integrating the area under peaks unique to each element. Quantification was performed using CasaXPS software (CasaXPS Version 2.3.15, Casa Software Ltd, http://www.casaxps.com/) using a smart background and standard peak type.

High resolution XPS spectra were acquired and smoothed in OriginPro (version 2019b) using second order polynomial Savitzky-Golay smoothing. The C1s, P2p, and Ca2p spectra were smoothed with a 15, 25, and 35 points of window, respectively. Further, the C1s, P2p, and Ca2p XPS spectra were Shirley baseline subtracted in Fityk (version 1.3.1).

The C1s spectra were also peak fit in Fityk using procedures previously described^24,25^. Specifically, C1s spectra were fit using Gaussian peak shapes with a fixed full-width-at-half-maximum of 1.4 eV. The peaks for carbon-containing functional groups consisted of C–P, C–C/C=C, C–O, C=O, and O–C=O that were centered at 283.5, 284.8, 286.5, 287.4, and 289.0 eV, respectively with a variance of ± 0.2 eV. The area under the fitted peaks in the high resolution C1s spectra is reported in atomic percent.

### Matrix printing

Matrices were printed by Dimension Inx (Chicago, IL). CaPG powder was sent to Dimension Inx, and Dimension Inx used its patented 3D printing technology to print high CaPG content matrices. Details of the ink formulation and print process have been reported in the literature^64,65^. The final product was a 3D matrix of 90 wt% CaPG and 10% poly(lactic-*co*-glycolic acid) (PLGA).

Through its research and development, Dimension Inx has identified matrix print parameters that are well suited for in vitro and in vivo applications. For this study, we purchased large sheets of each type (Supplementary Fig. 3) from which we were able to punch out matrices of desired size for experiments.

The in vitro matrix sheet was 3 cm × 3 cm × 0.1 cm. It consisted of 4 layers, of which each layer was 170 μm thick. The layers were 90° offset. The strut-to-strut distance was 700 μm. The spacing between adjacent fibers in same layer was 500 μm, and the strut diameter was 200 μm.

The in vivo matrix sheet was 3 cm × 3 cm × 0.05 cm. It consisted of 3 layers, of which each layer was 170 μm thick. The layers were 120° offset. The strut-to-strut distance was 700 μm. The spacing between adjacent fibers in same layer was 500 μm, and the strut diameter was 200 μm.

### Matrix bulk density

The bulk density (ρ_bulk_) of 3DP-CaPG matrices was determined by the matrix mass (*m*_matrix_) by volume (*V*_matrix_):

1 $${\rho }_{bulk}={m}_{matrix}/{V}_{matrix},$$

where *m*_matrix_ was measured on an analytical balance. The *V*_matrix_ was calculated using the thickness and diameter of the cylindrical matrices that were measured with calipers.

### Matrix total porosity

The total porosity (ϕ_total_) of 3DP-CaPG matrices, expressed as a percent, was calculated using the following:

2 $${\phi }_{total}=\frac{{\rho }_{T}-{\rho }_{bulk}}{{\rho }_{T}}\times 100\%,$$

where ρ_T_ is the theoretical density. The ρ_T_ was calculated using the rule of mixtures, which is a weighted average based on the composition of the material. In the case of 3DP-CaPG matrices, 90% is comprised of CaPG and 10% is PLGA that have theoretical densities of 2.26 g cm^–3^ and 1.30 g cm^–3^, respectively. Thus, the theoretical density of 3DP-CaPG matrices was calculated by the following:

3 $${\rho }_{T} = 0.9{\rho }_{graphite}+0.1{\rho }_{PLGA}=2.16\, \left[\text{g cm}^{-3}\right].$$

### Dynamic mechanical testing

All mechanical testing was conducted on a Discovery Hybrid Rheometer (TA Instruments, New Castle, DE) using a disposable, aluminum Peltier cylinder with a diameter of 25 mm as the bottom geometry and a sandblasted Peltier cylinder with a diameter of 8 mm as the top geometry. All measurements were acquired after applying a compressive 0.1 N pre-force.

Frequency sweeps were acquired in compression at room temperature with an axial strain of 0.3%. Data was acquired in triplicate for each disk over a frequency of 0.1–11.0 Hz with 10 points per decade. The storage and loss moduli of 3DP-CaPG matrices were reported at 1.0 Hz.

The stress–strain curves of the 3DP CaPG disks were measured in compression with a constant linear rate of 0.01 mm/s. The acquired data was not processed with a correction formula and is reported as-acquired. The ultimate compressive strengths (*UCS*) and maximum compressive strains (σ_F_) were determined from the peak inflection on the stress–strain curves indicative of material failure. Toughness (*U*_T_) was calculated from the area under the stress–strain curves.

### Cell culture conditions

Adipose–derived human mesenchymal stem cells (hMSCs) were purchased from Thermo Fisher Scientific (#R7788–115). The hMSCs were cultured in a humidified, 37 °C, 5% CO_2_ incubator (HERAcell 150i CO2 incubator with copper chamber, Thermo Fisher Scientific, #51026283) in filter cap 25 cm^2^ flasks (Greiner Bio-One CELLSTAR, #690175). “Growth media” is a commercially available formulation (Thermo Fisher Scientific) for the expansion and preservation of potency of hMSCs. Growth media consists of reduced-serum MesenPRO RS Medium (#12746012) that is supplemented with l-glutamine (#25030081) at a final concentration of 2 mM. Growth media was also spiked with penicillin/streptomycin (#15140122) at a final concentration of 100 U mL^–1^. “Osteogenic media” is a commercially available formulation (Thermo Fisher Scientific): StemPro Osteogenesis Differentiation Kit (#A1007201). Osteogenic media was also spiked with gentamicin (#15710064) diluted to 5 µg mL^–1^. For subculture, hMSCs were detached using TrypLE Express without phenol red (#12604013), as it participates in fewer non–specific reactions, decreasing cellular damage. No hMSC reagents contained phenol red since it affects osteogenic differentiation^86^. To ensure potency, hMSCs were not used beyond passage seven.

### hMSCs on matrices

For experiments, we chose to use cylindrical matrices of a 3.5 mm diameter. This size ensured the ability to acquire reliable, valid data while not requiring an excessive amount of material or hMSCs. Further, it matched the diameter of the calvarial defects used in the mouse model. Cylindrical matrices were punched out of a large (3 cm × 3 cm × 0.1 cm) sheet that was 3D printed using a biopsy punch. The matrices were placed into individual wells of 48 well tissue culture plates (Greiner Bio-One CELLSTAR, #677180) and sterilized based on methods described by the manufacturer and reported in literature^64,65^. The matrices were bathed in 70% ethanol for 1 h. Then, they were washed three times with cell-culture grade phosphate buffered saline (PBS, Thermo Fisher Scientific, #10010049) for at least 5 min per wash. Matrices were maintained in PBS until the hMSCs were ready for seeding.

Based on literature reports for similarly 3D-printed matrices^65^ and manufacturer (Dimension Inx) recommendations, we targeted a seeding density of ~ 5000 cells mm^–3^ to be administered in a seeding volume of 10 μL. This seeding density corresponded to ~ 50,000 cells matrix^–1^. To seed the cells onto the matrices, the matrices had the PBS aspirated, yielding matrices that were moist but not too wet. Then, 5 μL of 25,000 cells 5 µL^–1^ were directly pipetted on top of the matrices. After 25 min, the matrices were carefully inverted, and another 5 µL of 25,000 cells 5 µL^–1^ were directly pipetted onto the current tops. After a total of 50 min from the initial seeding (25 min from the second seeding), the wells containing the matrices were flooded with 0.500 mL of growth media.

Since it was inevitable that some cells would not adhere to the matrix but instead pass through the matrix and adhere to the tissue culture plate, after 1 day the matrices were carefully transferred using a sterile spatula to a pristine well with fresh media. This prevented any interaction and confounding effects from cells initially seeded on the tissue culture plate. Also, at this point, osteogenic media was first given to matrices that were designated to be cultured in it. Cells were cultured on the matrices for 10 days, and media changes were provided no longer than every 3.5 days.

### Cytocompatibility

After 10 days of the hMSCs being cultured on the matrices, the cell culture media was aspirated, the matrices gently washed with PBS, and then the cells were exposed to staining solution. Staining solution was added at 0.500 mL well^–1^ and consisted of PBS spiked with Hoechst 33342 at 20 µM (Thermo Fisher Scientific, #62249); Calcein AM at 5 µM (PromoKine, #PK–CA707-80011-2); and propidium iodide at 2 µM of propidium iodide (Alfa Aesar, #J66584). Cells were exposed to the staining solution for 15 min in the incubator. Then, the staining solution was removed, being replaced with fresh PBS.

Since the matrix is not only 3D but also highly absorbing due to the graphenic component, fluorescent microplate measurements would be inaccurate. Thus, to more accurately determine cytocompatibility, the matrices were directly imaged (Thermo Fisher Scientific, EVOS FL Auto Cell Imaging System, #AMAFD1000) with a 10×, 0.30 numerical aperture objective (Thermo Fisher Scientific, #AMEP 4681) and a 20×, 0.40 numerical aperture objective (Thermo Fisher Scientific, #AMEP4682). Hoechst 33342 labels the DNA of all cells (imaged with the DAPI light cube; Ex: 357/44 nm, Em: 447/60 nm; Thermo Fisher Scientific, #AMEP4650), and propidium iodide labels the DNA of dying cells whose membrane integrity are compromised (imaged with the RFP light cube; Ex: 531/40 nm, Em: 593/40 nm; Thermo Fisher Scientific, #AMEP4652). Imaging can be used to identify and quantify labeled nuclei without relying on quantification directly from fluorescence intensity. Thus, Hoechst 33342 and propidium iodide-labeled nuclei (> 740 per sample) were quantified from multiple, representative images of each matrix. Calcein AM is converted to a fluorescence form inside metabolically active cells (imaged with the GFP light cube; Ex: 470/22 nm, Em: 510/42 nm; Thermo Fisher Scientific, #AMEP4651). Average cellular viability (live cells as a percentage of all cells) were calculated for each matrix, and the individual matrices were then averaged together (*n* = 4).

To create images for display purposes, the as acquired fluorescence images were processed in Leica Application Suite Advanced Fluorescence Lite software 2.6.0 build 7266 (Leica Microsystems, https://www.leica-microsystems.com/). Individual images were overlaid and colormap ranges were adjusted to best visualize labeling.

### Alkaline phosphatase (ALP) expression

ALP expression was determined using the ImmPACT Vector Red Alkaline Phosphatase kit (Vector Laboratories, Inc., #SK-5105) based on the manufacturer’s protocol. After 10 days of hMSCs being cultured on the 3D printed matrices, the medias were aspirated, and the matrices were washed with PBS. Next, the hMSCs on the matrices were fixed by exposure to 3.7% formaldehyde in PBS v/v for 10 min. Then, the formaldehyde solution was aspirated, and the cells were washed with PBS. To each well, 500 µL of staining solution was added. The staining solution consisted of ImmPACT Vector Red substrate working solution spiked with Hoechst 33342 at a final concentration of 20 µM. The hMSCs were exposed to the staining solution for 1 h. Then, the samples were washed with PBS for 5 min and were then maintained in excess (500 µL) PBS.

Since the 3D printed matrices contain a significant amount of CaPG, the matrices are highly absorbing due to the graphenic backbone of CaPG. Thus, while the ALP assay can usually be quantified using a fluorescence microplate reader, these graphenic matrices would not yield reliable data. Thus, instead, the matrices were subjected to fluorescence and light microscopy. Using an automatic microscope (EVOS FL Auto Cell Imaging System) with a 10× objective, whole-matrix images of Hoechst 33342 fluorescence, ImmPACT Vector Red-labeled ALP fluorescence, and transmission bright field images were acquired. To probe the cellular/sub–cellular distribution of ALP expression at higher resolution, higher-magnification imaging was performed using a 20× objective and a long–working distance 40×, 0.65 numerical aperture objective (Thermo Fisher Scientific, #AMEP4683). Overlays of the different channels were created using Leica Application Suite Advanced Fluorescence Lite software. As-acquired large images that extended beyond the matrices (to ensure that entire matrices were imaged) were cropped to the size of the matrices in ImageJ (US National Institutes of Health).

To quantify ALP expression, the whole-matrix images of fluorescently labeled ALP were background intensity thresholded in ImageJ. Then, the images were read into MATLAB (The MathWorks, Inc.), which was used to calculate the sum of all the pixel intensity values. Multiple matrices were averaged together (*n* = 3), and quantified results are reported as sample mean plus and minus sample standard error of the mean.

### Alizarin red S (ARS) labeling

After 10 days of hMSCs being cultured on matrices, calcium deposits were labeled using ARS. A staining solution was prepared by diluting ARS (VWR, #97062-616) to 40 mM in DI water. To label the hMSCs, the cell culture media was aspirated; the matrices were washed with calcium-free PBS, the samples were fixed in 3.7% formaldehyde for 10 min; the formaldehyde solution was aspirated; the samples washed twice with PBS; and then the matrices were exposed to the ARS staining solution for 1.5 h at room temperature. After labeling, the samples were washed 7 times with PBS and then maintained in excess (~ 500 µL) PBS.

Whole matrix images were acquired using an automated microscope (EVOS FL Auto Cell Imaging System) with a 10× objective and a color camera. To quantify ARS labeling, the color whole-matrix images were read into MATLAB. The RGB format images were converted to CIELAB color space (L*a*b*). In L*a*b* color space, the a* channel represents the green/red opponent colors, where 0 is neutral, increasingly negative is increasingly green, and increasingly positive is increasingly red. Thus, the greater the value of a*, the more red. The mean value of a* was calculated for each matrix. Multiple matrices were averaged together (*n* = 3), and quantified results are reported as sample mean plus and minus sample standard error of the mean.

### Gene expression

To quantify osteogenic gene expression, reverse transcription quantitative polymerase chain reaction (RT-qPCR) experiments were designed and performed. Details are described below in accordance with the Minimum Information for Publication of Quantitative Real-Time PCR Experiments (i.e., MIQE) guidelines^87^.

To do so, hMSCs were cultured on 3D printed matrices—2 matrices for each type of media—for 10 days. Then, RNA was immediately extracted using a commercially available RNA purification kit (RNeasy Mini Kit, Qiagen, #74104). During the process, the lysates from the same media type were pooled together. Once the RNA was purified, it was assessed with a microvolume UV–Vis spectrophotometer (NanoDrop One, Thermo Fisher Scientific, #ND-ONE-W). Spectra from 190 to 800 nm with a 0.5 nm step size were acquired from 2 µL of RNA solution. Analysis of spectra indicated a strong absorption of guanidine HCl that was a leftover reagent from the RNA extraction/purification kit (Supplementary Fig. 5a). In our other studies, attempts at RNA cleanup to remove the guanidine HCl were unsuccessful but yielded viable RNA for RT-qPCR experiments. Thus, spectroscopic analysis of RNA purity was not feasible. To obtain a rough estimate of the concentration of RNA, we used the generally accepted RNA extinction coefficient of 40 ng cm µL^–1^ and calculated concentration using the Beer-Lambert equation, as absorption from guanidine HCl was relatively low at 260 nm. RNA solution was stored at − 20 °C until use.

All PCR plastic consumables were certified DNase, RNase, and pyrogen free, and the pipette tips contained aerosol filters and were purchased sterilized via γ-irradiation. All PCR procedures were performed in a sterile environment. RT-qPCR experiments were setup on ice in 96–well PCR plates (VWR, #82006-644) and sealed (VWR, #60941-070) before beginning RT.

TaqMan hydrolysis probes (Thermo Fisher Scientific) were used to fluorescently report amplification in real time. All probes spanned exon junctions, negating the need for a DNA digestion step. All probes used were commercially available through Thermo Fisher Scientific: bone morphogenetic protein 2 (BMP-2, #Hs01055564_m1); collagen type I alpha 1 (COL1A1, #Hs00164004_m1), runt-related transcription factor 2 (RUNX–2, #Hs00231692_m1); small nuclear ribonucleoprotein D3 (SNRPD3, #Hs00188207_m1); and proteasome subunit beta 2 (PSMB2, Hs00267650_m1). Amplicon lengths were 84, 66, 116, 68, and 80 base pairs for BMP–2, COL1A1, RUNX–2, SNRPD3, and PSMB2, respectively. SNRPD3 and PSMB2 were selected as reference genes as they recently have been shown to be exceptionally uniformly expressed^88^.

To perform RT-qPCR on the RNA, a one-step assay (TaqMan RNA-to-C_T_ 1-Step Kit, #4392938, ThermoFisher Scientific) was used. Master mixes were made for all components as appropriate, and the reaction volume was 10 µL. For a typical individual reaction, there was 5.00 µL of TaqMan RT-PCR Mix (2×), 0.50 µL of the TaqMan hydrolysis probe (20× mix diluted to final concentrations of the forward primer, reverse primer, and probe of 900, 900, and 250 nM, respectively), 0.25 µL of TaqMan RT enzyme mix (40×), 2 µL of RNA solution (~ 10 ng RNA), and 2.25 µL of DPEC-treated water (Thermo Fisher Scientific, #AM9906). All samples were run in quadruplicate. To minimize competition for PCR resources, there was no multiplexing.

Reverse transcription/cDNA synthesis and real-time PCR was performed on an Applied Biosystems 7300 instrument controlled by Sequence Detection Software Version 1.4 (https://www.thermofisher.com/order/catalog/product/4379633). The reverse transcription step occurred at 48 °C for 15 min and was followed by activation of AmpliTaq Gold DNA Polymerase Ultra Pure at 95 °C for 10 min. Then 60 cycles were performed of denaturing at 95 °C for 15 s and anneal/extend at 60 °C for 1 min. Sequence Detection Software was used to calculate and plot ΔRn and to automatically calculate a baseline and threshold to determine the threshold cycle, also more generally referred to as quantification cycle (C_q_).

To determine PCR efficiency, serial tenfold dilutions of the RNAs were performed, and the samples were subjected to RT-qPCR for SNRPD3. The C_q_ values were identified, and for those more than 3 C_q_ units smaller than the no template control, they were plotted versus the logarithm of RNA concentration (Supplementary Fig. 5b). When plotted in this manner, the slope of the line is a measure of efficiency: $Efficiency={10}^{\left(-1/slope\right)}-1$. A PCR efficiency of 100% corresponds to a slope of − 3.32. To determine PCR efficiency of the experimental samples, a line was fit to the data, and the slope was used to calculate PCR efficiency. PCR efficiencies for the undiluted RNA samples were acceptable and had well-resolved C_q_ values. Thus, these were the amounts of RNA used for the RT-qPCR experiments.

The C_q_ values for the RNA samples for BMP-2, RUNX-2, and PSMB2 were not less than 3 away from their corresponding C_q_ values of the no template control. Thus, those samples were not valid. However, the COL1A1 and SNRPD3 yielded accurate data as the no template controls either had a much later amplification (i.e., larger C_q_ value) or had no detectable amplification at all.

To analyze and report gene expression, the ${2}^{-\Delta \Delta {C}_{T}}$ method was used^89^. COL1A1 was the target; SNRPD3 was the reference; a PCR efficiency of 100% was assumed for all experiments, and the growth media sample is the calibrator. Four measurements were made (from the pooled RNA of two matrices) for each sample. The sample means and standard errors of the means were calculated, and then the uncertainties were propagated using the derivative method of error propagation for calculations on the data.

### Mechanical properties

After 10 days of hMSCs being cultured on the matrices, the mechanical properties of the matrices were analyzed using axial compressive dynamic mechanical analysis (DMA) on a TA Instruments Discovery Hybrid 2 rheometer with a DMA attachment. Individual matrices were delicately removed from the cell culture media by gently scooping with a spatula and placing them on the bottom of a parallel plate geometry. The top plate was lowered until an axial force of ~ 0.1 N, corresponding to a prestress of ~ 10 kPa, was achieved. Then, the samples were subjected to axial compressive DMA using a strain of 0.3% and a frequency sweep from 0.1 to 11 Hz. A total of four different matrices for each media condition were analyzed. Averaged data is presented as both the entire frequency sweep, as well as bar plots of storage (*E'*) and loss (*E''*) moduli at 1 Hz for ease of comparison. Data is sample mean, and error bars are standard error of the mean.

### Animal study

The objective of this study was to evaluate the bone regenerative capacity of 3DP-CaPG in a mouse non-union calvarial defect model. This study was a randomized, blinded, and controlled laboratory experiment. All aspects of the animal protocol were approved by UConn Health Institutional Animal Care and Use Committee (IACUC), animal welfare assurance number D16-00295, and all methods were performed in accordance with the relevant guidelines and regulations. The study was conducted in accordance with ARRIVE guidelines. All mice were constructed in the laboratory of Dr. David Rowe at UConn Health and were generated, bred, and maintained at the Center for Laboratory Animal Care of UConn Health. The animals had free access to sterile water and standard rodent chow ad libitum.

CD-1 transgenic mice containing the 3.6-kb fragment of the rat collagen type 1 promoter fused to a cyan fluorescent protein (Col3.6Cyan) were used as the donor mice to isolate bone marrow stromal cells. NOD.Cg-Prkdc^scid^ Il2rg^tm1Wj1^/SzJ (NOD *scid* gamma, NSG) immunodeficient mice containing the 3.6-kb fragment of the rat collagen type 1 promoter fused to a topaz fluorescent protein (NSG/Col3.6Topaz) were used as host mice. The mice were divided into three groups: 3DP-G, 3DP-CaPG, and OsteoWrap demineralized cortical plate membrane (demineralized bone matrix, DBM). There were no inclusion or exclusion criteria beyond mortality resulting from infectious disease, as described below. During the allocation and conduction of the experiment, the authors were blinded. The authors only became aware of each group’s experimental conditions during analysis. The matrices for implantation were prepared using disposable biopsy punches of 3.5 mm diameter and sterilized as described for in vitro experiments.

Since these mice are immunocompromised, some occasionally become sick and die. These mortality events are due to their immunocompromised state and not the experimental conditions. Thus, if and when mice became sick and died during the course of the experiment, subsequent experiments were conducted to ensure that at least 4 valid replicates were obtained for each condition to ensure significant results. Ultimately, the final sample sizes were as follows: DBM (+) BMSCs: *n* = 6; DBM (−) BMSCs: *n* = 4; 3DP-G (+) BMSCs: *n* = 6; 3DP-G (−) BMSCs: *n* = 5; 3DP-CaPG (+) BMSCs: *n* = 5; and 3DP-CaPG (−) BMSCs: *n* = 5.

### Bone marrow stromal cell isolation and culture

Col3.6Cyan (female, 8–10 weeks old) were used to derive bone marrow stromal cells. The mice were sacrificed by CO_2_ asphyxiation followed by cervical dislocation. The femurs and tibias were carefully isolated and dissected from the surrounding soft tissue. The two ends were cut and the bone marrow was collected by flushing complete media consisting of high glucose DMEM with l-glutamine, 10% FBS, 1% penicillin/streptomycin with a 25 gauge needle. When all the marrows were obtained, the suspension was passed through an 18.5 gauge needle. The cells were then counted and plated in a 100 mm dish at a density of approximately 6 × 10^7^ cells per dish. The cells were kept in a Sanyo incubator under low oxygen conditions. At day 3 and 6, the media was replaced with fresh complete media.

At day 7 of BMSC culture, the cells were washed with PBS, detached with Accutase, and resuspended in complete media at a concentration of 1 × 10^6^ cells mL^–1^. The cells were centrifuged at 1200 rpm for 5 min, after which the media was removed completely. The cells were resuspended in 10 μL of media, from which 5 μL was taken and seeded onto the matrices that were placed onto glass slides. After 1 h, the matrices were flipped, and the remainder of the cell solution was seeded onto the other side. The cells were allowed to attach for another hour, prior to implantation.

### Surgical procedure

NSG/Col3.6Topaz (male, 11–13 week old) were used for calvarial surgeries. The mice were anesthetized with an intraperitoneal injection of Ketamine (135 mg kg^–1^)/Xylazine (15 mg kg^–1^). The head was shaved and the surgical site was cleaned with 75% ethanol. An incision was made just off the sagittal midline to expose the parietal bone. A 3.5 mm defect was created on one side of non-suture associated parietal bone using a trephine drill. The calvarial disk was carefully removed to avoid any injury to the underlying dura mater and the matrices were implanted into the defect. The skin was sutured with 5-0 vicryl, and the mice were subcutaneously injected with 0.08 mg kg^–1^ buprenorphine for analgesia. An additional dose of buprenorphine was given within 24 h of surgery.

### Gross morphology and radiology

One day prior to sacrifice, Alizarin Red (AC) bone label at a dose of 30 mg kg^–1^ body weight was injected intraperitoneally to mark areas of newly deposited mineral within 24 h of sacrifice.

Animals were sacrificed at 8 weeks post-implantation by CO_2_ asphyxiation followed by cervical dislocation. The intact calvaria were carefully dissected from the skull and surrounding tissue and fixed in 10% formalin at 4 °C. After 2–3 days, the calvaria were imaged digitally and next radiographically (6 s at 26 kVp) using a digital capture X-ray cabinet (Faxitron LX-60).

### Histological analysis

After X-ray imaging, the calvaria were placed in a 30% sucrose solution in PBS (pH 7.4) for 1 day. The tissues were then positioned in Richard-Allan Scientific™ Neg-50™ frozen section medium (Thermo Scientific). Cryosections (5 μm) through the non-decalcified calvaria were obtained on a Leica CM3050-S cryostat (Leica, Wetzlar) using a disposable steel blade (Thermo Scientific) and tape transfer process (cryofilm type IIC (10), Section-Lab Co. LTD) as previously described. The slides were then prepared with 50% glycerin in PBS as the mounting medium. The sections were initially imaged for differential interference contrast (DIC) using the Zeiss Axio Scan.Z1 (Carl Zeiss Microscopy). Next, the endogenous fluorescence of the Col3.6Topaz and Col3.6Cyan fluorescent reporters, and the AC mineralization label were imaged. The sections were then sequentially stained and imaged for TRAP enzymatic activity, ALP, DAPI, and toluidine blue O, as previously reported^24,78,90^. This sequence was possible because the cryofilm tape adheres to the tissue and allows for the coverslip to be removed between the imaging steps without damaging the section.

### Statistics

Data is reported as mean ± standard error of the mean, unless otherwise indicated. Differences are labeled as statistically significant if the two-tailed *p*-value, calculated using an unpaired *t*-test (Prism, GraphPad Software, https://www.graphpad.com/), is less than 0.05. The *p*-values for growth media compared to osteogenic media for cellular viability is 1, for ALP expression is 0.22, for ARS labeling is 0.0070, for COL1A1 expression is 0.030, for *E'* is 0.21, and for *E''* is 0.17. The *p*-values for *E'* and *E''* for the pristine matrices compared to matrices with hMSCs cultured on them for 10 days are 0.75 and < 1 × 10^–4^, respectively.

## Supplementary Information

Supplementary Figures.
