# Supplementary material for: Ultra-low binder content 3D printed calcium phosphate graphene scaffolds as resorbable, osteoinductive matrices that support bone formation in vivo
Source: Sci Rep. 2022 Apr 28;12:6960. doi: 10.1038/s41598-022-10603-3 (PMC9050648; doi:10.1038/s41598-022-10603-3)
Supplement: Supplementary file 1 — Supplementary Figures. [file 41598_2022_10603_MOESM1_ESM.pdf]

Supplementary Information

Ultra-low binder content 3D printed calcium phosphate graphene scaffolds as resorbable, osteoinductive matrices that support bone formation in vivo

Leila Daneshmandi[1,2,3,4,10], Brian D. Holt[5,10], Anne M. Arnold[5,6], Cato T. Laurencin[1,2,3,4,7,8*] & Stefanie A. Sydlik[5,9*]

[1] Connecticut Convergence Institute for Translation in Regenerative Engineering, UConn Health, Farmington, CT 06030, USA
[2] Raymond and Beverly Sackler Center for Biological, Physical and Engineering Sciences, UConn Health, Farmington, CT 06030, USA
[3] Department of Biomedical Engineering, University of Connecticut, Storrs, CT 06269, USA
[4] Department of Orthopaedic Surgery, UConn Health, Farmington, CT 06030, USA
[5] Department of Chemistry, Carnegie Mellon University, 4400 Fifth Avenue, Pittsburgh, PA 15213, USA
[6] Current Address: National Security Directorate, Pacific Northwest National Laboratory, Richland, WA 99354, USA
[7] Department of Material Science and Engineering, University of Connecticut, Storrs, CT 06269, USA
[8] Department of Chemical and Biomolecular Engineering, University of Connecticut, Storrs, CT 06269, USA
[9] Department of Biomedical Engineering, Carnegie Mellon University, 5000 Forbes Avenue, Pittsburgh, PA 15213, USA
[10] These authors contributed equally: Leila Daneshmandi, Brian D. Holt
* email: laurencin@uchc.edu; ssydlik@andrew.cmu.edu

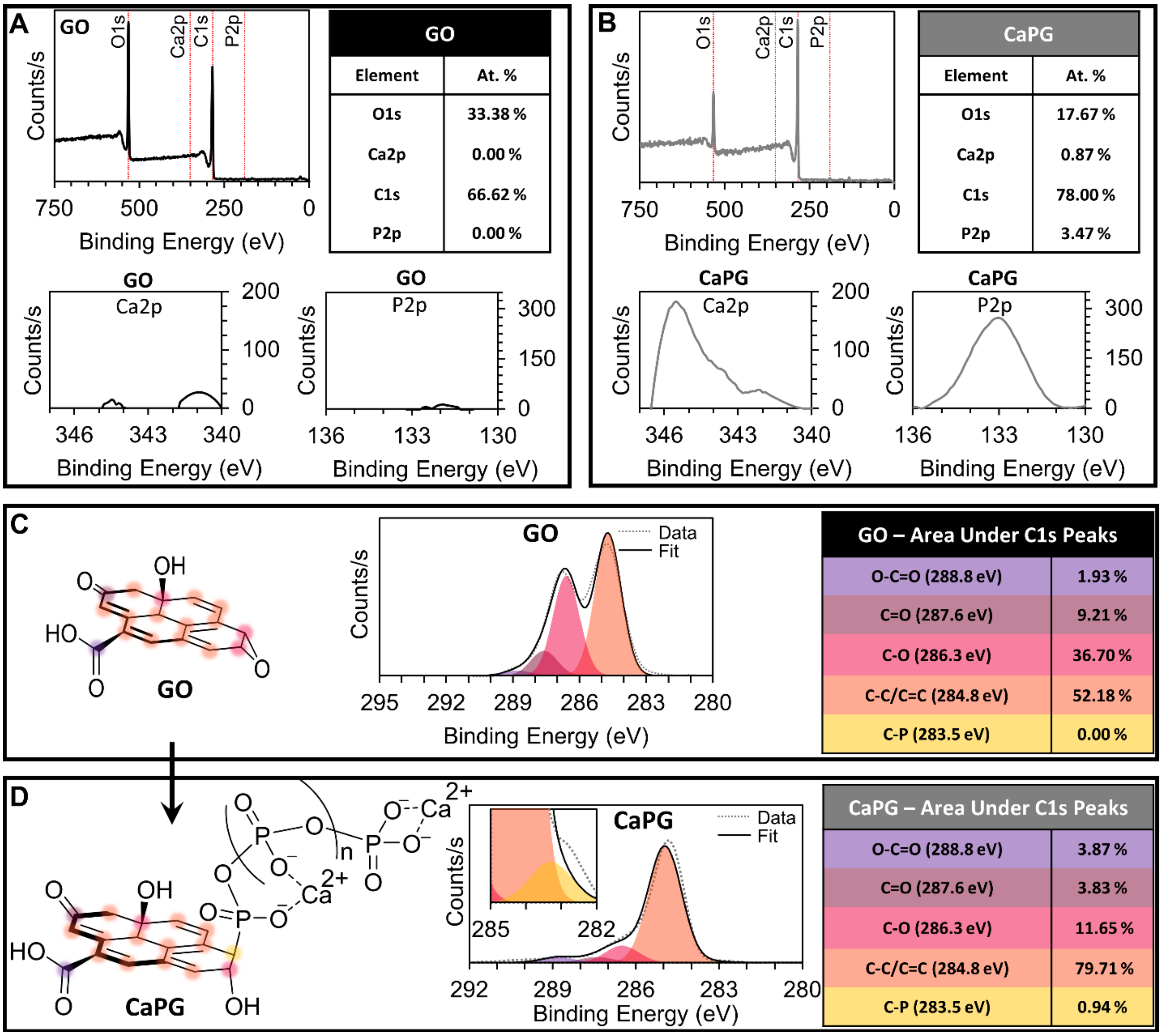

Supplementary Fig. 1 X-ray photoelectron spectroscopy (XPS) of graphene oxide (GO) and calcium phosphate graphene (CaPG) powders. a GO and **b** CaPG elemental survey scans with characteristic element peaks highlighted in red; table of element quantification from the red labelled peaks in the survey scans; high-resolution calcium (Ca2p) spectra; and high-resolution phosphorus (P2p) spectra. **c** GO and **d** CaPG representative chemical structures with chemically distinct carbons color coded to match the peaks in the peak-fit high-resolution carbon (C1s) spectra, and quantification of the area under the peaks (in atomic percent) represented in tables. Note that the graphenic backbone of GO and CaPG in panels (**c**,**d**) are represented as pyrene structures for simplicity.

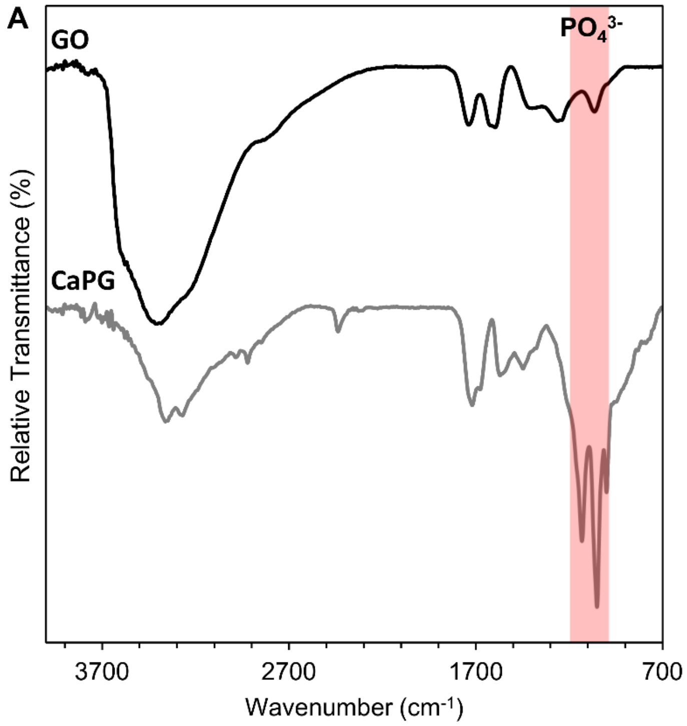

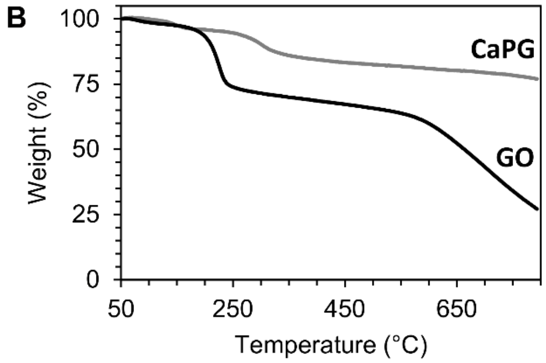

C

TGA Parameters	GO	CaPG
T_o (°C)	203.7	270.9
T_e (°C)	236.4	326.5
T_p (°C)	223.8	302.9
%D	16.3	6.1
Char Wt. %	27.2	77.0

Supplementary Fig. 2 FTIR spectroscopy and TGA characterization. a Fourier transform infrared (FTIR) spectra of graphene oxide (GO) and calcium phosphate graphene (CaPG) powders. The characteristic phosphate (PO_4^{3-}) stretches at 1200–1000 cm^{-1} are highlighted in red. **b** Thermogravimetric analysis (TGA) thermograms of GO and CaPG. **c** The characteristic, TGA degradation parameters for GO and CaPG. Parameters include the onset temperature (T_o), first derivative peak temperature (T_p), endset temperature (T_e), and total weight percent loss (%D) of degradation. The char weight percent (Char Wt. %) is also reported, which is the remaining weight percent at 800 °C. Note that the T_o, T_e, %D, and Char Wt. % were measured from thermograms in panel B. The T_p were determined from the first derivative curves (not shown) of the TGA thermograms at the maximum peak inflection during degradation. Note that all parameters correspond to the first degradation event for GO and CaPG.

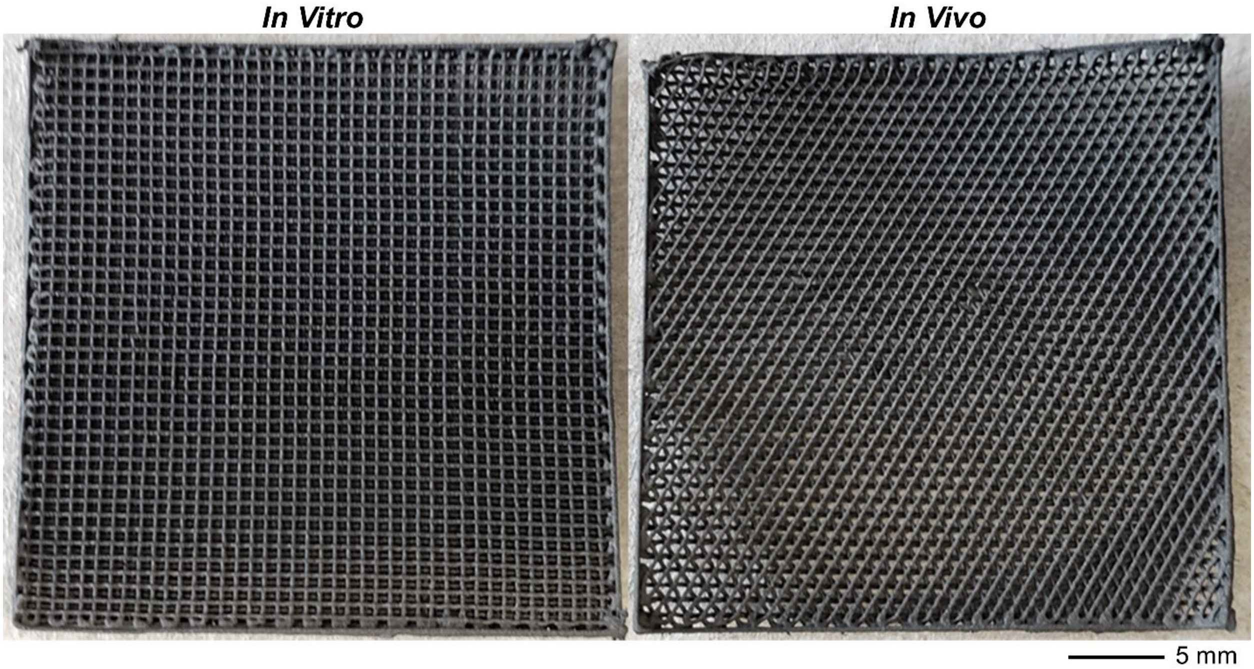

Supplementary Fig. 3 Optical images of the as-produced in vitro and in vivo matrix sheets.

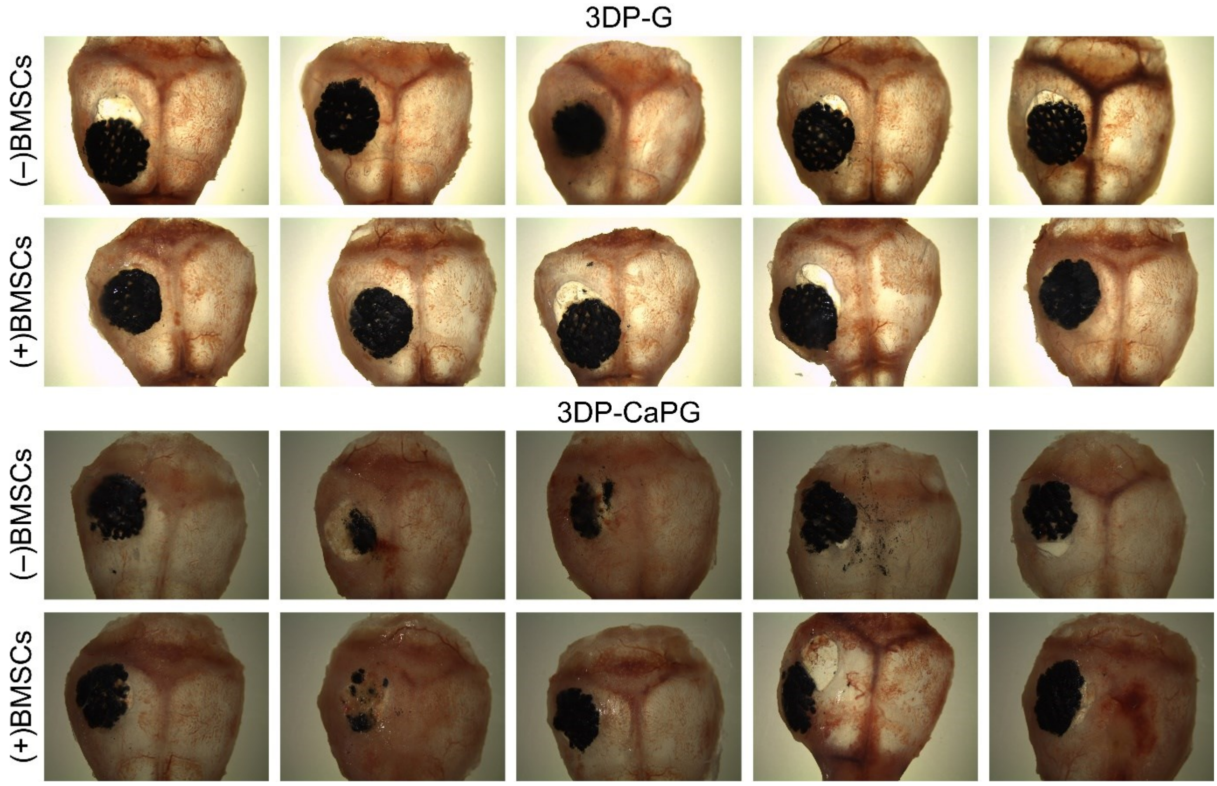

Supplementary Fig. 4 Images of the calvarial defect site 8 weeks after implantation.

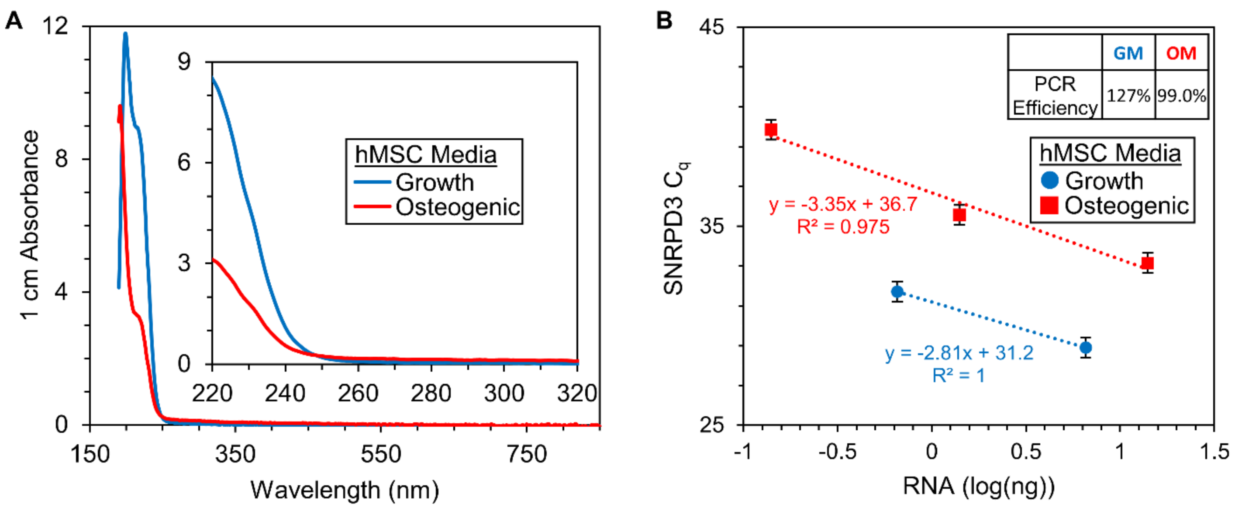

Supplementary Fig. 5 RT-qPCR analysis. a Absorbance spectra of the RNA solutions. The spectra show strong absorption from guanidine HCl that masks the contributions from the RNA. **b** Determination of PCR efficiency through the use of a serial dilution standard curve. The valid range of RNA amount was limited due to the presence of PCR inhibitors at high concentrations of RNA and detected signal from the no template control samples at large C_q values. Experimental samples were only run with RNA concentrations within the valid range determined here. The higher than ideal PCR efficiency for the growth media sample is likely due to PCR inhibitors, as the presence of PCR inhibitors is known to produce efficiencies > 110%.
